# Supplementary material for: Reconstruction of ovine axonal cytoarchitecture enables more accurate models of brain biomechanics
Source: Commun Biol. 2022 Oct 17;5:1101. doi: 10.1038/s42003-022-04052-x (PMC9576772; doi:10.1038/s42003-022-04052-x)
Supplement: Supplementary file 2 — Supplementary Information [file 42003_2022_4052_MOESM2_ESM.pdf]

Reconstruction of Ovine Axonal Cytoarchitecture Enables More Accurate Models of Brain Biomechanics

Andrea Bernardini[1,*✉], Marco Trovatelli[2,*], Michał M. Kłosowski[3], Matteo Pederzani[4], Davide Danilo Zani[2], Stefano Brizzola[2], Alexandra Porter[3], Ferdinando Rodriguez y Baena[1], Daniele Dini[1,✉]

[1] Department of Mechanical Engineering, Imperial College London, London SW7 2AZ, UK
[2] Faculty of Veterinary Medicine, Università degli Studi di Milano Statale, 26900 Lodi, Italy
[3] Department of Materials, Imperial College London, London SW7 2AZ, UK
[4] Department of Electronics, Information and Bioengineering, Politecnico di Milano, 20133 Milan, Italy

* These authors have equally contributed to the article.
✉ a.bernardini16@imperial.ac.uk, ✉ d.dini@imperial.ac.uk

Supplementary Information

Supplementary Methods

Data Repository

A database[1] containing all images acquired for the ovine brain samples analysed in this investigation, the detailed codes to perform the 3D reconstruction and measurements of individual axons and the data post-processing has been created and made available via open access.

A detailed description of the steps taken to obtain the data presented in the main article and further information and graphical outputs are reported below with relevant links to the data sets and codes made available to the interested reader.

3D Measurements: Acquisition

Centerline Input Parameters: MIMICS software allows the extraction of an array of measurements from the fitted centerline. To create centerline a smoothing factor, a resolving resolution and a distance between control points is manually input:

- The smoothing removes noises, via a Fourier smoothing method, from the first computation of the centerline. The smoothing threshold value depends on the level of detail needed and therefore, from the goodness of the segmentation manually executed. In the occurrence of extremely twisted 3D entities and sharp turns in the geometry, the algorithm may fail to compute the centerline within the 3D volume. Lowering the smoothing factor prevented these occurrences. The reasonable homogeneity of the axonal structures and cytoarchitecture allowed the choice of a single value per each volume, although it has been occasionally adjusted from axon to axon.
- The resolving resolution dictates the size of the axons that are recognized by the algorithm. The smaller the value, the smaller the tubular structures recognized.

This value was set after a fast overview of the axons present in each volume and the smallest axonal diameter detected was used as resolving resolution.
- The distance between control points was set to be the interlayer distance which is the acquisition depth of the FIB-SEM imaging phase (150nm)

Centerline Output Parameters: For each centerline, .txt files were exported with the data extrapolated from the centerline itself. The measurements written on the file are arranged in columns of a length equal to the number of control points where measurements have been taken. The typical file consists of many outputs, in this study only the following have been used:

- Best-Fit diameter
- Cross-sectional Area
- Ellipticity

3D Reconstruction and Measurements: Post-Processing

Via a series of Matlab scripts, the measurements have been imported, analyzed and the output stored as input for the creation of .stl models.

A first Matlab script skims through the columns and imports only the quantities of interests into Matlab structures. This *importing_data.m* script can be found commented in the folder "*03_scripts*" of the database[1].

Each axon has been measured over its length every 0.15 μm, therefore for each axon a certain number of measurements, corresponding to the slices representing the axon, has been acquired. Therefore, per each axon, a mean value for cross-sectional area, a mean value for best-fit diameter and a mean value for ellipticity was calculated over each axonal length. A statistical analysis was performed only on the mean cross-sectional area values, being a dependency between area, best-fit diameter and ellipticity variables. Firstly, the few outliers found in the data were filtered via the median absolute deviation filtering method implemented in Matlab via the function *rmoutliers*. This procedure was done for each subject (04, 05, 06), for each area (CC, CR, FO). Then, three non-parametric tests Kruskal-Wallis, with a p-value fixed at 0.05, were performed on the three brains to check for statistically relevant differences between the areas CC, CR and FO. For brain 04 (see figure S1a) an extremely significant difference was found between the areas (*p-value=0*); for brain 05 (see figure S1b) a very significant difference was found between the areas (*p-value=0.0006*); and for brain 06 (see figure S1c) also a significant difference was registered (*p-value=0.0112*).

Then, to create the most representative database that would comprise the measured real life variation between subjects, all the data from the three subjects were grouped together by CC (figure S2a), CR (figure S2b) and FO (figure S2c) and then furtherly analyzed to define the probability distribution function (pdf) that underlies the data from each area. Following previous histological studies,[2–4] normalized histograms, representing empirical pdf, were plotted against the plots of the lognormal pdf. The pdf was itself calculated on the CC, CR and FO data with the *fitdist* Matlab function by the means of maximum likelihood. The location parameter (μ) and the shape parameter (σ) characterizing the pdf were measured for CC, CR and FO. These couples of parameters are what is then used for the 3D reconstruction algorithm.

3D in silico RVEs Generation: Algorithm

A series of Matlab scripts were created to recreate the 3D geometries for further software for analysis (*e.g.* F.E.A.). The algorithm ensures periodicity in the x-y direction and recreates the measured tortuosity of the axons in the z direction. The flowchart in figure S3 is a simplified version of the algorithm used for the generation of the virtual 3D RVEs; the full commented code can be found in the folder "*03_scripts*" of the database[1].

Supplementary Figures

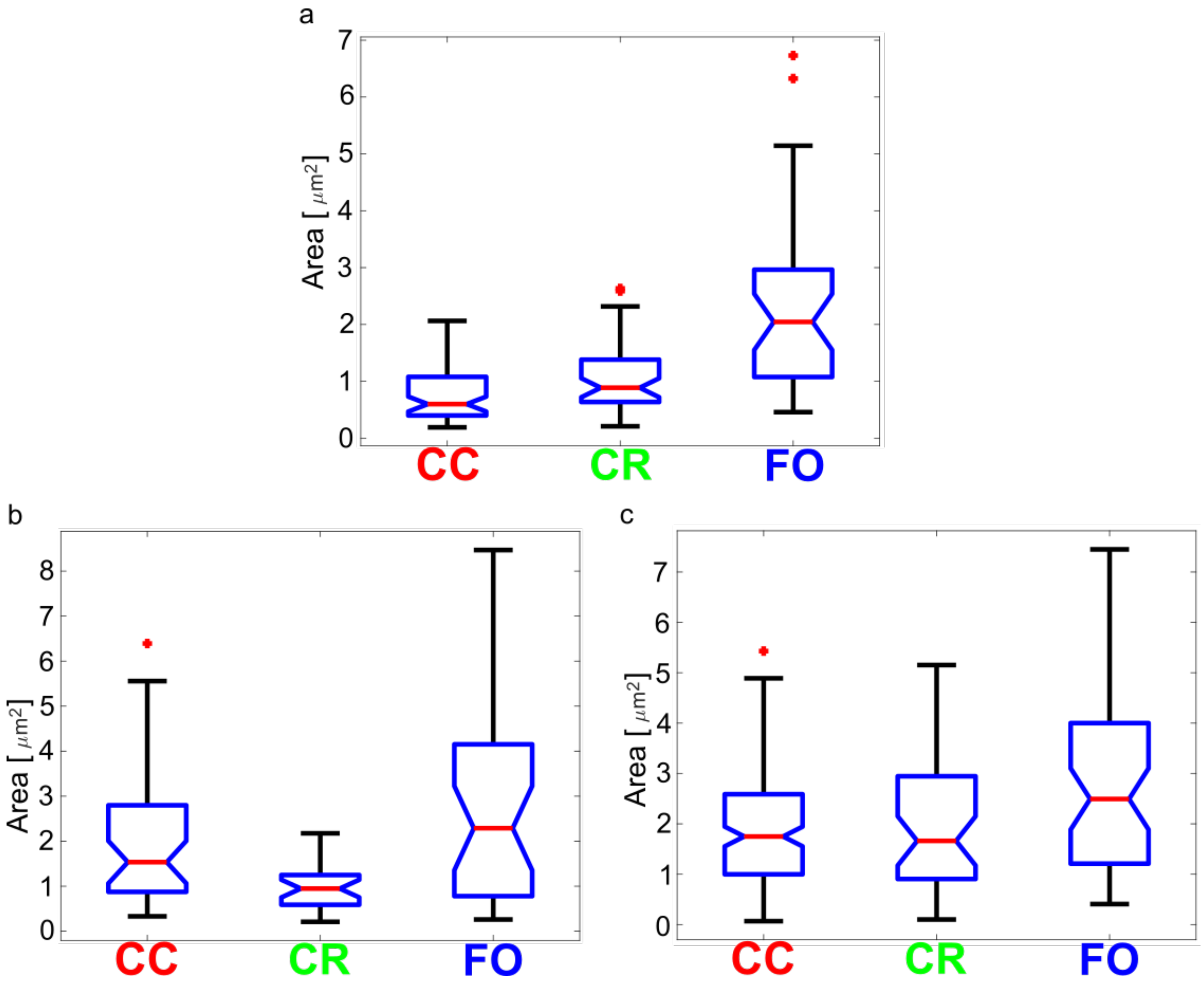

Figure S1|Boxplots of the average axonal cross-sectional areas for each axonal tract per each brain. a, Brain 04 shows a very significant difference, with the FO showing the most dissimilar behavior among the three axonal tracts. CC and CR both show a small interquartile range (IQR) and median values comprised within the interval 0.5 and 1 μm^2. FO exhibits a much larger IQR than the other two areas with a higher median value nearly reaching 2 μm^2. **b,** Brain 05 shows the CC and CR being more similar to each other than to the CR. CC has a bigger IQR than CR but with a median nearer to its lower quartile. CR is characterized by a very small IQR and an equidistant median from the upper and lower IQR boundaries. FO shows a bigger spread of data with a median value higher than 2 μm^2. **c,** Brain 06 has a significant difference between the areas but they all appear more homogeneous in their IQR, although the FO still exhibits the largest IQR among the three. Additionally, the median values all range between 1.5 and 2.5 μm^2.

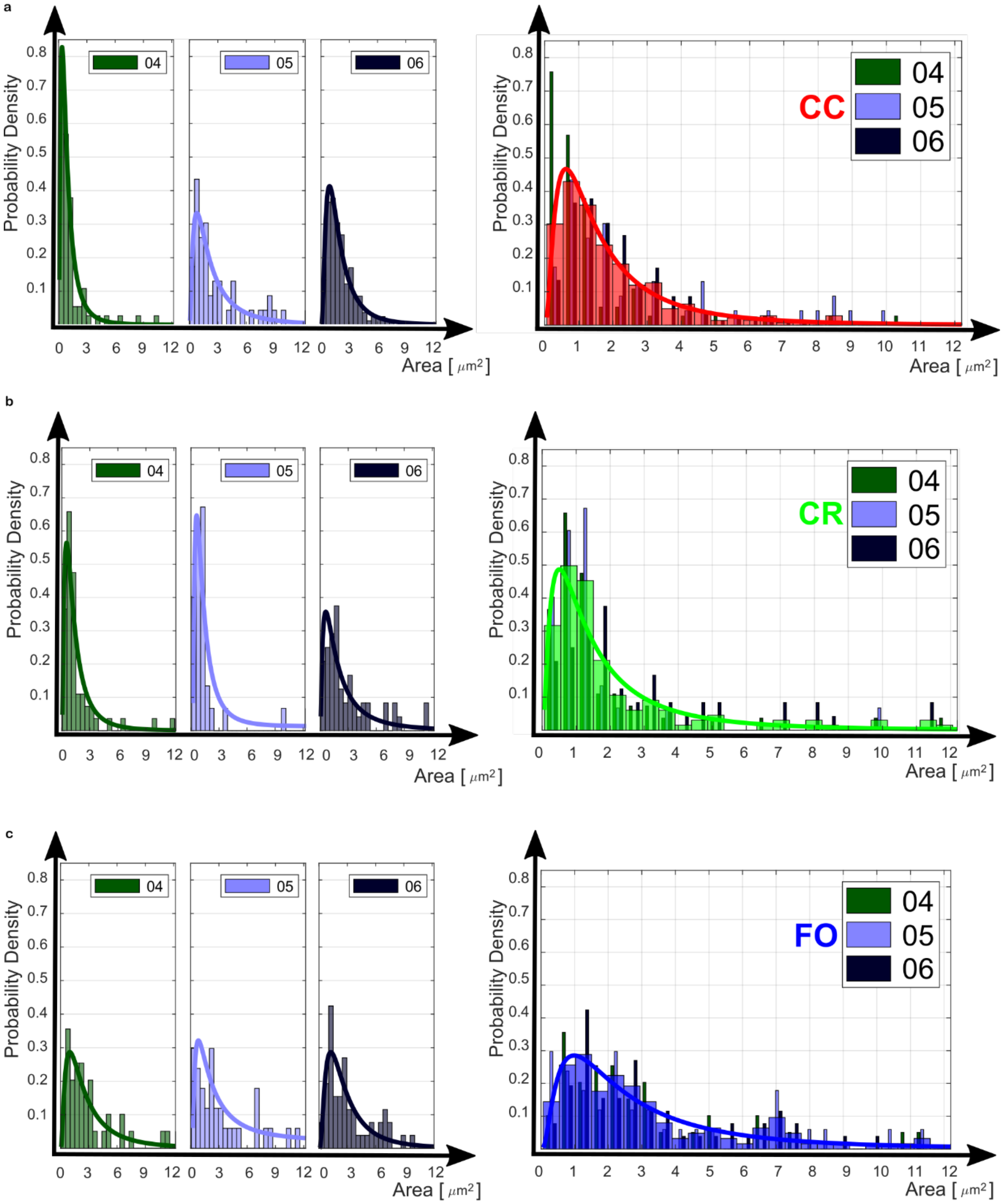

Figure S2|Checking the underlying pdf. a, The CC shows agreement for all subjects in the location parameter, however for subject 04 a prominent skewness to the left is recorded, hinting at a higher quantity of smaller axons detected. The overall CC distribution shows that most of the axons in the CC have a cross sectional area ranging between 0.5 and 3 μm^2. **b**, The CR shows all the subjects with the location parameter within the 0.5-3μm^2 but the subject 06 seems to have more instances with a higher value of cross-sectional area. However, the overall CR distribution is not too different from the overall CC. **c**, The three subjects show a very similar behavior of the FO with no prominent differences between them. Additionally, all of them present much thicker axons when compared to the other two areas. The overall distribution presents a location parameter μ within the same range of the other two areas but its different shape parameter (σ) describes a bigger spread of the cross-sectional areas showing the existence of bigger axons in the FO.

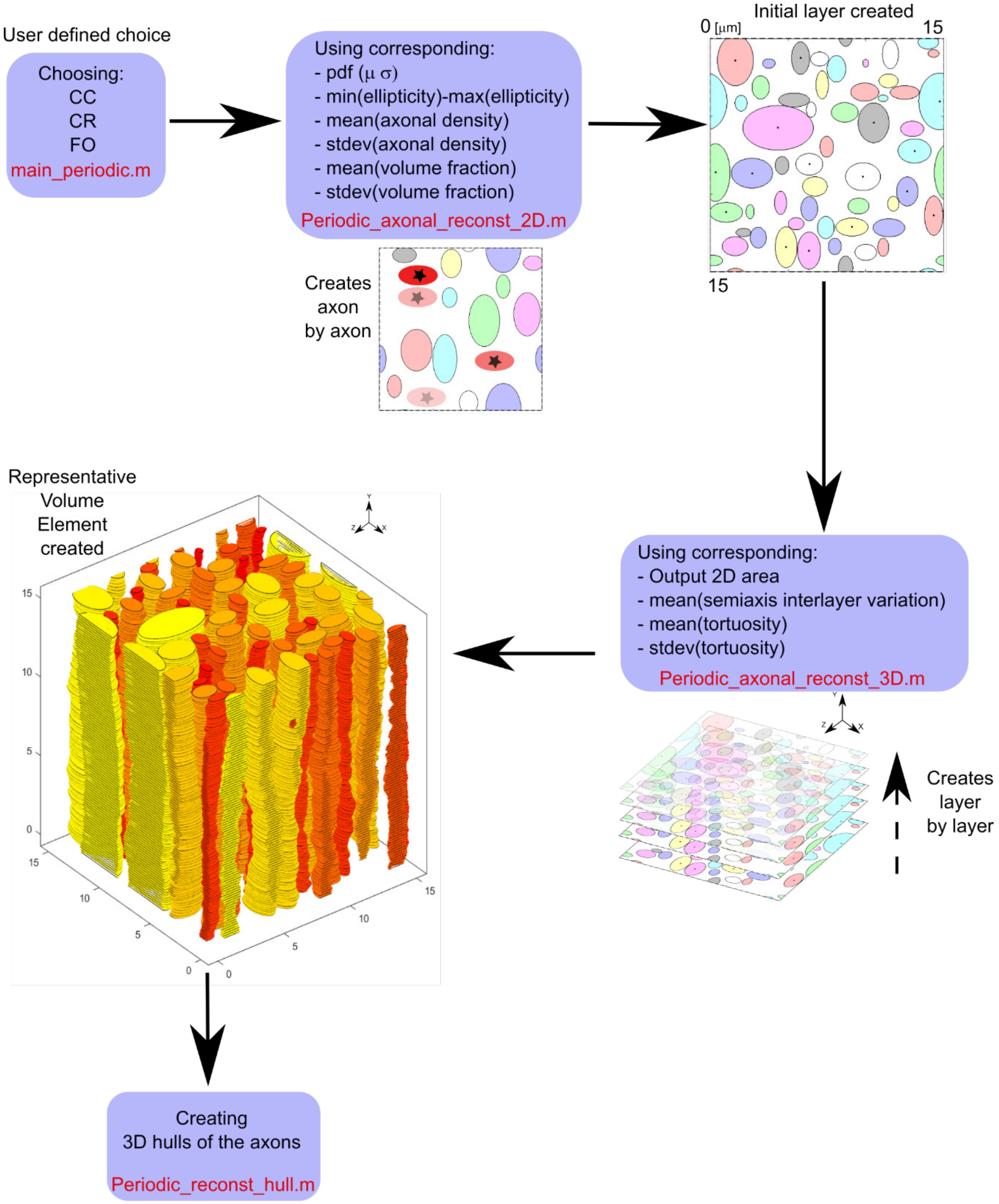

Figure S3| Schematic representation of the 3D reconstruction algorithm. The scheme represents the functioning of the three scripts that allow the creation of ever-different RVE of CC, CR and FO. The volumes, although always different are always representative of the observed data. In fact the input parameters are randomly chosen by the code within the acceptable range of variation observed in the experimental parameters.

Supplementary References

1. Bernardini, A., Trovatelli, M. & Dini, D. EDEN2020 Ovine Brain Dataset for Imaging and Reconstruction of the Cytoarchitecture of Axonal Fibres (Version 1.0) [Data set]. *Zenodo* (2021) doi:10.5281/zenodo.4772440.

2. Liewald, D., Miller, R., Logothetis, N., Wagner, H. J. & Schüz, A. Distribution of axon diameters in cortical white matter: an electron-microscopic study on three human brains and a macaque. *Biol. Cybern.* **108**, 541–557 (2014).

3. Caminiti, R., Ghaziri, H., Galuske, R., Hof, P. R. & Innocenti, G. M. Evolution amplified processing with temporally dispersed slow neuronal connectivity in primates. *Proc. Natl. Acad. Sci.* **106**, 19551–19556 (2009).

4. Sepehrband, F. *et al.* Parametric Probability Distribution Functions for Axon Diameters of Corpus Callosum. *Front. Neuroanat.* **10**, 1–9 (2016).
