# Supplementary material for: Reconstruction of ovine axonal cytoarchitecture enables more accurate models of brain biomechanics
Source: Commun Biol. 2022 Oct 17;5:1101. doi: 10.1038/s42003-022-04052-x (PMC9576772; doi:10.1038/s42003-022-04052-x)
Supplement: Supplementary file 3 — Description of Additional Supplementary Files [file 42003_2022_4052_MOESM3_ESM.pdf]

Description of Additional Supplementary Files

File name: Supplementary Data 1
Description: The source data behind the graphs (figures) in the paper.
