# Supplementary material for: Reconstruction of ovine axonal cytoarchitecture enables more accurate models of brain biomechanics
Source: Commun Biol. 2022 Oct 17;5:1101. doi: 10.1038/s42003-022-04052-x (PMC9576772; doi:10.1038/s42003-022-04052-x)
Supplement: Supplementary file 5 — Reporting Summary [file 42003_2022_4052_MOESM5_ESM.pdf]

Corresponding author(s): Professor Daniele Dini

Last updated by author(s): Sep 18, 2022

Reporting Summary

Nature Portfolio wishes to improve the reproducibility of the work that we publish. This form provides structure for consistency and transparency in reporting. For further information on Nature Portfolio policies, see our Editorial Policies and the Editorial Policy Checklist.

Statistics

For all statistical analyses, confirm that the following items are present in the figure legend, table legend, main text, or Methods section.

n/a	Confirmed	
☐	☒	The exact sample size (n) for each experimental group/condition, given as a discrete number and unit of measurement
☐	☒	A statement on whether measurements were taken from distinct samples or whether the same sample was measured repeatedly
☐	☒	The statistical test(s) used AND whether they are one- or two-sided<br>*Only common tests should be described solely by name; describe more complex techniques in the Methods section.*
☒	☐	A description of all covariates tested
☐	☒	A description of any assumptions or corrections, such as tests of normality and adjustment for multiple comparisons
☒	☐	A full description of the statistical parameters including central tendency (e.g. means) or other basic estimates (e.g. regression coefficient) AND variation (e.g. standard deviation) or associated estimates of uncertainty (e.g. confidence intervals)
☐	☒	For null hypothesis testing, the test statistic (e.g. F, t, r) with confidence intervals, effect sizes, degrees of freedom and P value noted<br>*Give P values as exact values whenever suitable.*
☒	☐	For Bayesian analysis, information on the choice of priors and Markov chain Monte Carlo settings
☒	☐	For hierarchical and complex designs, identification of the appropriate level for tests and full reporting of outcomes
☒	☐	Estimates of effect sizes (e.g. Cohen's d, Pearson's r), indicating how they were calculated

Our web collection on statistics for biologists contains articles on many of the points above.

Software and code

Policy information about availability of computer code

Data collection	This is fully detailed in the Supplementary Information file and the Zenodo repository provides the source codes used.
Data analysis	This is fully detailed in the Supplementary Information file and the Zenodo repository provides the source codes used.

For manuscripts utilizing custom algorithms or software that are central to the research but not yet described in published literature, software must be made available to editors and reviewers. We strongly encourage code deposition in a community repository (e.g. GitHub). See the Nature Portfolio guidelines for submitting code & software for further information.

Data

Policy information about availability of data

All manuscripts must include a data availability statement. This statement should provide the following information, where applicable:

- Accession codes, unique identifiers, or web links for publicly available datasets
- A description of any restrictions on data availability
- For clinical datasets or third party data, please ensure that the statement adheres to our policy

A data availability statement has been included. All data and codes made available via a Zenodo repository: Bernardini, A., Trovatelli, M. & Dini, D. EDEN2020 Ovine Brain Dataset for Imaging and Reconstruction of the Cytoarchitecture of Axonal Fibres (Version 1.0) [Data set]. Zenodo (2021) doi:10.5281/zenodo.4772440.

Field-specific reporting

Please select the one below that is the best fit for your research. If you are not sure, read the appropriate sections before making your selection.

☒ Life sciences ☐ Behavioural & social sciences ☐ Ecological, evolutionary & environmental sciences

For a reference copy of the document with all sections, see nature.com/documents/nr-reporting-summary-flat.pdf

Life sciences study design

All studies must disclose on these points even when the disclosure is negative.

Sample size	See Methods and Supplementary Information
Data exclusions	See Methods and Supplementary Information
Replication	Multiple ovine brains were used
Randomization	Not relevant to the study
Blinding	Blinding was not applicable to the study as samples had to be selected from specific areas of ovine samples

Reporting for specific materials, systems and methods

We require information from authors about some types of materials, experimental systems and methods used in many studies. Here, indicate whether each material, system or method listed is relevant to your study. If you are not sure if a list item applies to your research, read the appropriate section before selecting a response.

Materials & experimental systems

n/a	Involved in the study	
☒	☐	Antibodies
☒	☐	Eukaryotic cell lines
☒	☐	Palaeontology and archaeology
☐	☒	Animals and other organisms
☒	☐	Human research participants
☒	☐	Clinical data
☒	☐	Dual use research of concern

Methods

n/a	Involved in the study	
☒	☐	ChIP-seq
☒	☐	Flow cytometry
☒	☐	MRI-based neuroimaging

Animals and other organisms

Policy information about studies involving animals; ARRIVE guidelines recommended for reporting animal research

Laboratory animals	See details in Methods; ovine brains were included in this study, carried on in the context of the EU's Horizon EDEN2020 project (https://www.eden2020.eu/). Three healthy, female ovis aries (1 year old, 70 Kg weight) have been used for this study. All animals have been treated under the European Communities Council Directive (2010/63/EU), adhering to the laws and regulations on animal welfare enclosed in D.L.G.S. 26/2014 and it has been approved by the Italian Health Department with authorization n° 635/2017. After culling via intravenous potassium chloride overdose, following the authorization n° 635/2017, the cerebrum has been removed.
Wild animals	*Provide details on animals observed in or captured in the field; report species, sex and age where possible. Describe how animals were caught and transported and what happened to captive animals after the study (if killed, explain why and describe method; if released, say where and when) OR state that the study did not involve wild animals.*
Field-collected samples	*For laboratory work with field-collected samples, describe all relevant parameters such as housing, maintenance, temperature, photoperiod and end-of-experiment protocol OR state that the study did not involve samples collected from the field.*
Ethics oversight	Italian Health Department with authorization n° 635/2017.

Note that full information on the approval of the study protocol must also be provided in the manuscript.
